# Establishment of efficient hypocotyl-derived protoplast isolation and its application in soybean (*Glycine max* [L.] Merr.)

**DOI:** 10.3389/fpls.2025.1587927

**Published:** 2025-05-20

**Authors:** Kihwan Kim, Junseop Shin, Jeong-Dong Lee, Won-Chan Kim

**Affiliations:** ^1^ Department of Applied Biosciences, Kyungpook National University, Daegu, Republic of Korea; ^2^ Upland Field Machinery Research Center, Kyungpook National University, Daegu, Republic of Korea; ^3^ Department of Integrative Biology, Kyungpook National University, Daegu, Republic of Korea

**Keywords:** *Glycine max*, hypocotyl-derived protoplast, PEG-Ca^2+^ mediated transfection, protoplast yield, transient gene expression

## Abstract

Soybean is important crop species in agriculture, food science, and biotechnology due to their valuable components. The exploration of soybean genetic traits is being highlighted for the advancement of research in various aspects. The utilization of plant biotechnology, plant protoplast techniques, for the study of genetic characteristics is being extended to various agricultural crop species. The quintessential goal of genetic characterization utilizing plant protoplasts encompasses the provision of stable plant protoplasts alongside the establishment of transfection condition. Despite the numerous studies on protoplast isolation, standardized and reliable soybean protoplasts protocols for comprehensive investigations into the intricate regulatory mechanisms governing immune responses, cellular processes, and developmental pathways remain insufficiently established. In this study, we propose an efficient methodology for the protoplast isolation and the PEG-Ca^2+^ mediated transfection of soybean [*Glycine max* (L.) Merr.] cultivar (Williams 82). The protoplast isolation entailed the evaluation of variables including mannitol concentration, enzyme mixture composition, and enzymatic digestion duration. The optimal conditions for hypocotyl-derived protoplast isolation were identified as 0.4 M mannitol, an enzyme mixture containing 1.5% (w/v) cellulase and 0.4% (w/v) macerozyme, and an 8-hour enzymatic digestion period, resulting in high viability and protoplast yield (>3.0 × 10^6^/g FW). For the PEG-Ca^2+^ mediated transfection process, the parameters assessed including PEG concentration, plasmid quantify or purified recombinant proteins, and PEG-Ca^2+^ incubation duration. The validation of the reliability of hypocotyl-derived protoplast system through transient gene expression demonstrates its utility as a robust platform for analysis of genetic traits in soybean. This could extend the scope of application to understanding the cell-to-cell interactions for physiological responses in soybean.

## 1 Introduction

Soybean (*Glycine max* [L.] Merr.), a legume crop, contains high protein and fat content along with an abundance of vitamins and minerals, rendering them a globally popular food resource (Erdman and Fordyce, 1989; Singh et al., 2008; Hammond and Jez, 2011; Baianu et al., 2012). Moreover, Soybean are attracting attention due to their potential applications in various fields, responding to the escalating demands for active compounds and eco-friendly materials. Isoflavones derived from soybean have garnered attention for their potential as active compounds in antioxidants (Devi et al., 2009; Tyug et al., 2010), skin aging prevention (Back et al., 2018; Kim, 2021), and hormone regulation (Duncan et al., 1999; Persky et al., 2002). Polyurethane foam formulated from soybean oil extract has been developed into high-performance materials, such as insulation materials (Tan et al., 2011; Dhaliwal et al., 2019), making it a sustainable material. Plant cells regulate sophisticated regulatory mechanisms through cell-to-cell interactions (Hake and Char, 1997; Dupuy et al., 2008; Thibivilliers and Libault, 2021). Accordingly, it is important to conduct research that characterizes genes and reveals their structural and functional properties considering the interaction between environmental factors and plant cell processes.

Plant biotechnology utilizing protoplasts from which plant cell walls have been enzymatically removed is an ideal platform for investigating genetic characteristics in various plant species (Chen et al., 2006; Yoo et al., 2007; Zhang et al., 2011; Pitzschke and Persak, 2012; Li et al., 2018). Protoplast-based systems offer expanded methodologies for the study of gene function through the exploration of temporally responsive signaling and metabolic pathways that elicit physiological responses (Sheen, 2001; Lehmann et al., 2020; Gilliard et al., 2021). Moreover, transient gene expression constitutes a rapid and efficient technique that complements the limitations inherent in other technique for studying gene function in plant species that are difficult to genetic modification. Recently, the scope of application of platform using plant protoplasts for CRISPR/Cas9 ribonucleoprotein (RNP)-mediated genome editing and single-cell transcriptomics is expanding. The utilization of CRISPR/Cas9 RNP facilitates precise modifications at the genomic levels (Subburaj and Zanon Agapito-Tenfen, 2023; Kim et al., 2024), while single-cell transcriptomics provides insight into gene expression profiling of individual cells (Ryu et al., 2019; Rich-Griffin et al., 2020; Xu et al., 2021). These advantages have led to focusing on the development of protoplast isolation and transient gene expression system aimed at improving the efficiency of protoplast-based systems in variety of plant species. Although mesophyll-based protoplast isolation methods from many model plant and crop species are well-established such as Broccoli (Yang et al., 2022), Cymbidium (Ren et al., 2020), Cucumis (Huang et al., 2013), Ginkog (Han et al., 2023), Saccharum (Wang et al., 2021), Solanum (Wang et al., 2022), Petunia (Kang et al., 2020), and Pinellia (Tian et al., 2023), procuring sufficiently viable protoplasts from mature leaves of many plant species remain a challenge due to variances in cell composition among different plant species. The establishment and application of protoplast-based system hinge upon the availability of stable, viable protoplasts and the determination of suitable transfection condition.

Soybean [*Glycine max* (L.) Merr.] cultivar (Williams 82) has been completely sequenced, serve as a standardized genetic model for investigating gene function and the physiological responses of soybeans to environmental stress, leveraging a wealth of genetic information (Schmutz et al., 2010; Li et al., 2017). The gene expression or genetic variation within QTL regions that play a major role influence local adaptation and soybean productivity within various environments (Han et al., 2012; Wang et al., 2015; Lin et al., 2021). The availability of the soybean genome sequence has facilitated the expeditious identification and functional analysis of genes, leading to significant advancements in deciphering regulatory mechanisms governing soybean responses to environmental signal (Lu et al., 2017). Nevertheless, the comprehensive exploration and validation of the fundamental roles of many genes in these regulatory mechanisms remain limited, primarily attributed to the challenges associated with soybean transformation techniques. Various technical limitations in soybean transformation, including genotype-dependent transformation constraints across different varieties (Altpeter et al., 2016; Kausch et al., 2019), as well as challenges in achieving successful plant regeneration through tissue culture, which are further complicated by stress-induced transcriptional reprogramming and epigenetic changes during protoplast isolation and culture, significantly impede progress (Pasternak et al., 2020). To efficiently discover the function of these genes, it is essential to adjustment a rapid and effective system for gene expression or protein interaction analysis by utilizing protoplast system (Lu et al., 2021). Overcoming these obstacles requires substantial investments in time-consuming and resources. Consequently, developing protoplast isolation and transient gene expression systems for the Williams 82, a cultivar with extensive genomic data available, is crucial for advancing our understanding of gene function and regulatory mechanisms in soybean. Despite the wide adoption of protoplast systems in various plant species, research progress in soybean protoplast isolation has been limited, with relatively few studies establishing reproducible and high-yield protocols. Although several studies have reported the isolation and utilization of soybean protoplasts (Zieg and Outka, 1980; Tricoli et al., 1986; Lin et al., 1987; Sun et al., 2015), most lack a comprehensive optimization of key parameters such as tissue type, enzyme composition, and digestion time.

Here, our research aims to establish efficient protoplast isolation and transfection conditions for the Willams 82. Based on the fact that the yield of high-yield viable protoplast is closely correlated to the combination of plant cell wall-degrading enzymes and osmotic pressure conditions, the yield of viable protoplasts was evaluated according to the combination of mannitol concentration, enzyme mixture composition, and enzymatic digestion duration. Additionally, the impact of transfection efficiency was evaluated based on the concentration of polyethylene glycol (PEG) concentration, plasmid DNA quantity or purified recombinant proteins, and PEG-Ca^2+^ incubation duration. We propose optimal conditions for protoplast isolation and transformation that could facilitate more effective genetic studies in the Williams 82.

## 2 Materials and methods

### 2.1 Plant materials and growth condition

Seed of soybean [*Glycine max* (L.) Merr.] cultivar (Williams 82) were sown in soil pots. The seeds were grown at 25°C under long-day conditions (LD, 16 h light/8 h dark photoperiod) in growth chamber. Light intensity was approximately 100 μmol m^− 2^ s^− 1^. The 2-week-old soybean seedlings were used for protoplast isolation.

### 2.2 Protoplast isolation

To assess the protoplast isolation efficiency, the method was modified established protocols (Jia et al., 2018; Ren et al., 2021; Kim et al., 2023a). 2 g of fresh weight soybean tissues were sliced into approximately 0.5 mm strips on sterile filter paper using a blade and immediately transferred to sterilized 9 cm petri dishes containing 10 mL of freshly prepared Enzyme solutions, which had been incubated at 55°C for 10 minutes prior to filtration through a 0.2 μm syringe filter. Subsequently, cell wall degradation was carried out at 25°C in dark with a rotation of 55 rpm for various enzyme treatment duration. The Enzyme solution containing protoplasts was diluted with an equal volume of W5 solution and then filtered through a 100 μm nylon mesh into a 50 mL conical tube. The retrieved protoplasts were overlaid on 10 mL of 0.6 M sucrose and then centrifuged at 100 × g for 10 min. The purified protoplasts at the sucrose-MMG solution (0.4 M mannitol, 15 mM MgCl_2_, 4 mM MES, pH 5.7) interface were transferred to a new round-bottom tube. The transferred protoplasts were rinsed twice in 1 mL MMG solution through centrifugating at 100 × g for 1 min. The details of the solutions used in this study are provided in **Supplementary Table S1**.

### 2.3 FDA assay

Fluorescein diacetate (FDA) dissolved in acetone (2 mg of FDA in 1 mL acetone) was utilized to visualize viable protoplasts through green fluorescence. The 10 μL of rinsed protoplasts was stained with 1 μL 0.2% (w/v) FDA solution, and then incubated at 25°C in dark for 2 min. The protoplast with green fluorescence were visualized by the Olympus BX53 fluorescence microscopy (Olympus, Japan).

### 2.4 Assessment of viable and visible protoplast yield

The viable protoplast yield was calculated following a minor modified protocol (Kang et al., 2020; Ren et al., 2020). The total number of isolated protoplasts was counted using a hemocytometer under the Lecia ICC50 HD optical microscopy (Leica, Germany). The viable protoplasts yield was determined as the total number of protoplasts with green fluorescence through FDA staining divided by the total number of observed protoplasts and then divided by the gram of fresh weight soybean tissues used for protoplast isolation (total number of protoplasts with green fluorescence/total number of observed protoplasts/g FW).

To achieve precise quantification of visible protoplast yield, the visible protoplast yield was normalized using UV-VIS spectrophotometric method. The optimal absorbance at optical density (OD_680 nm_) was determined by scanning the entire visible light spectrum to identify the most suitable OD value. A 100 μL aliquot of protoplasts suspended in 1 mL of MMG solution was further diluted in 900 μL of MMG solution and measured at OD_680 nm_ using a Ultrospec 8000 UV/VIS spectrophotometer (GE Healthcare Life Sciences, USA), and then the correlation between absorbance (OD_680 nm_) and visible protoplast yield was analyzed to generate a standard curve (**Supplementary Figure S1**).

### 2.5 Preparation of plasmid

To validate the transfection efficiency, plasmids for three distinct transient gene expression systems were constructed. First, the homemade plasmid, pTrTK-eGFP-BMLC, was constructed to determine the success of transfection. To construct the pTrTK-eGFP-BMLC, the nucleotides encoding the enhanced green fluorescent protein inserted between 2 × Cauliflower mosaic virus (CaMV) 35S constitutive promoter and the Nopaline synthase (NOS) terminator of the pTrTK-BMLC plasmid constructed in the previous study (Kim et al., 2023a).

Second, pTrTK-GmGATA58-eYFP-BMLC, was constructed to enable subcellular localization. It had been reported that the gene encoding the GmGATA58 transcription factor (GenBank accession number: XM_003550586) is in the cell nucleus (Zhang et al., 2015, 2020). The coding sequence (CDS) of the *GmGATA58* gene was obtained from trifoliate leaves of soybean through previous established protocols of total RNA extraction and first-strand cDNA synthesis (Kim et al., 2023b). The full-length CDS of *GmGATA58* was amplified through PCR utilizing specific primers designed to remove the stop codon. The amplified PCR products were digested with restriction enzyme (*Spe*I and *Xma*I) and then ligated in the pTrTK-eYFP-BMLC plasmid.

Third, pET28a-GUS and pET28a-NAN plasmid were constructed to assess recombinant protein activity in soybean protoplasts. The GUS reporter gene was amplified through PCR using specific primers targeting the GUS encoding sequences in the pTrGUS plasmid (Ko et al., 2009). The amplified PCR products were digested with restriction enzyme (*Bam*HI and *Sac*I) and then ligated in the bacterial expression vector pET28a(+) (Novagen, UK). The NAN reporter gene was amplified through PCR using specific primers targeting the NAN encoding sequences in the pTrNAN plasmid used in the previous study (Kim et al., 2021). The amplified PCR products were digested with restriction enzyme (*Bam*HI and *Sac*I) and then ligated in the bacterial expression vector pET28a(+) (Novagen, UK). The details of the primers used in this study are provided in **Supplementary Table S2**.

### 2.6 Recombinant protein expression and purification

The pET28a-GUS and pET28a-NAN plasmid was used to transform *Escherichia coli* (*E*. *coli*) strain BL21 (DE3). To overexpress the recombinant His_6_-GUS and His_6_-NAN protein, each single *E. coli* BL21 (DE3) introduced the pET28a-GUS and pET28a-NAN plasmid was cultured in LB broth containing 50 μg/mL kanamycin to a final OD_600nm_ of 0.5 at 37°C, and then IPTG(isopropyl-β-D-thiogalactopyranoside) was added and incubated at 16°C for 16 h.

For purification, cell pellets were resuspended in binding buffer [20 mM Tris-HCl, 500 mM NaCl, 5 mM imidazole, pH 7.9] and lysis brought about by sonication. Cell debris was removed by centrifugation (15,000 × g for 20 min at 4°C). and the recombinant His_6_-GUS and His_6_-NAN protein was isolated from the lysis supernatant using Ni^2+^-NTA (Qiagen, USA) affinity chromatography according to the manufacturer’s instructions (**Supplementary Figure S2**).

### 2.7 PEG-Ca^2+^ mediated transfection

To assess the efficiency of PEG-Ca^2+^ mediated transfection, we developed a through modified protocol based on previous studies (Wang et al., 2022; Han et al., 2023). The 10 μL each plasmid with different concentrations was gently mixed with 100 μL of rinsed protoplast (1.0 × 10^4^ protoplasts) in 2 mL round-bottomed Eppendorf tube, followed by addition of an equal volume (110 μL) of PEG-Ca^2+^ solution with different final concentration of PEG. Subsequently, the mixture was incubated at 25 °C in dark with various PEG-Ca^2+^ incubation duration. Two volumes (440 μL) of W5 solution were mixed to stop the transfection reaction followed by centrifugation at 100 × g for 1 min. The transfected protoplasts were rinsed twice in 440 μL W5 solution through centrifugating at 100 × g for 1 min and resuspending with 250 μL WI solution in 2 mL round-bottomed Eppendorf tube. The WI solution containing transfected protoplasts incubated at 25 °C in dark for 24 h. The details of the solutions used in this study are provided in **Supplementary Table S1**.

To determinate the PEG-Ca^2+^ mediated transfection, the pTrTK-eGFP-BMLC plasmid expressing the enhanced green fluorescent protein was transfected into rinsed protoplasts. The enhanced green fluorescent was visualized and photograph by using the Olympus BX53 fluorescence microscopy with green filter (Excitation wavelength: 480 nm; Emission wavelength: 535 nm).

To determinate the transfection efficiency of recombinant protein, 10 μL of purified recombinant His_6_-GUS and His_6_-NAN protein were co-transfected into protoplasts. The transfection efficiency of recombinant protein was determined by dividing the amount of 4-MU, a degradation product of 4-MUG or 4-MU-NANANa, degraded by transfected protoplasts by enzyme activity of each recombinant protein.

### 2.8 GUS and NAN activity assay

To determinate the GUS activity and NAN activity, the transfected protoplasts with each recombinant His_6_-GUS and His_6_-NAN protein was collected by centrifugation at 100 × g for 1 min. The collected protoplasts lysed by Lysis solution [25 mM Tris-HCl, 1 mM DTT, 2 mM DACTAA (trans-1,2-Diaminocyclohexane-N,N,N’N’-tetraacetic acid monohydrate), 10% (w/v) glycerol, 1% (w/v) Triton X-100] through vortexing for 1 min and centrifugating at 12,000 × g for 1 min. The 10 μL supernatant was transferred into the 100 μL 4-methylumbelliferyl-β-D-glucuronide (4-MUG) stock solution or 2-(4-methylumbelliferyl)-α-D-N-acetylneuraminic acid sodium salt (4-MU-NANANa) in Eppendorf tube and then incubate at 37°C in dark for 10 min. The reaction was stopped by adding 0.2 M Na_2_CO_3_ solution. The GUS activity was calculated by measuring 4-methylumbelliferone (4-MU), a decomposition product of 4-MUG. The NAN activity was calculated by measuring 4-MU, a decomposition product of 4-MU-NANANa. The 4-MU value was measured using a Specta Max Gemini XS (Molecular Devices, USA) with excitation wavelength at 355 nm and emission wavelength at 460 nm. Total protein concentration from transfected protoplasts was quantitatively analyzed using Bradford method (Bradford, 1976). The GUS and NAN activity were defined as picomoles 4-MU per milligram of protein per min (4-MU pmol/mg protein/min).

### 2.9 Subcellular localization

To detect whether the target protein is localized in cell nucleus, the transfected protoplasts with the pTrTK-GmGATA58-eYFP-BMLC plasmid were stained with 300 nM DAPI (4’,6-diamidino-2-phenylindole) dissolved in PBS buffer. DAPI signals and enhanced yellow fluorescent were visualized and photography by using the Olympus BX53 fluorescence microscopy with DAPI filter (Excitation wavelength: 375 nm; Emission wavelength: 460nm) and yellow filter (Excitation wavelength: 495 nm; Emission wavelength: 540 nm).

### 2.10 Statistical analysis

The data are presented as mean of at least three biological replications ± standard deviation (SD). All experiments were analyzed using one-way analysis of variance (ANOVA) using SAS v9.4 (SAS institute, USA). Multiple comparisons were performed using Tukey’s honestly significant difference test (*p* < 0.05).

## 3 Results

### 3.1 Effect of different tissues protoplast isolation efficiency in soybean seedling

**Figure 1:**
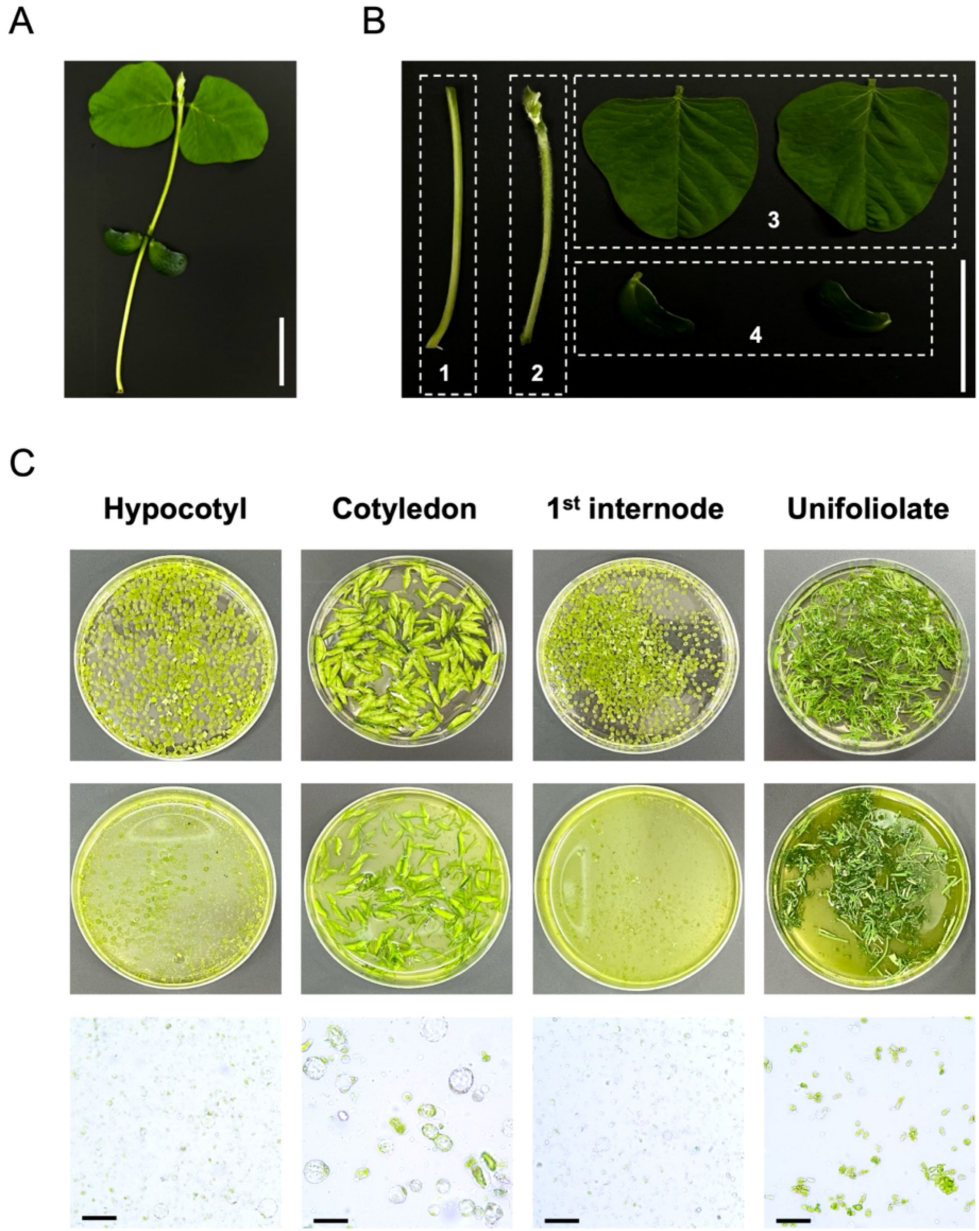
Isolation of protoplasts from different tissues of soybean seedlings [*Glycine max* (L.) Merr.] cultivars (Williams 82). **(A)** 2-week-old soybean seedlings. Scale bars = 5 cm. **(B)** Different tissues of soybean seedlings for preparation of protoplasts. Tissues were divided into four regions (1, hypocotyl; 2, 1^st^ internode; 3, unifoliolate; 4, cotyledon). Scale bars = 5 cm. **(C)** Isolation of soybean protoplasts from different tissues and enzymatic digestion process. Top: 9 cm petri dishes containing different tissues of soybean seedlings before enzymatic digestion. Middle: 9 cm petri dishes showing the results after enzymatic digestion. Bottom: The protoplast status in different tissues of soybean seedlings under bright-field of microscope. Scale bars = 50 μm.

To establish a versatile and highly viable soybean protoplast isolation system, the selection of appropriate soybean tissues is crucial for achieving high protoplast yields. To identify the most suitable tissues for efficient protoplast isolation, we isolated protoplasts from hypocotyls, cotyledons, 1^st^ internodes, and unifoliolate leaves (**Figure 1**). In unifoliolate leaves, enzymatic digestion was insufficient, resulting in inconsistent protoplasts morphology. This suggests that the presence of epicuticular waxes in unifoliolate leaves hindered the penetration of digestive enzymes, thereby negatively affecting the enzymatic digestion. In contrast, hypocotyls, cotyledons, and 1^st^ internode showed successful enzymatic digestion. In particular, the protoplasts isolated from hypocotyls showed the highest yield per unit fresh weight with consistent protoplast morphology. Therefore, hypocotyl was selected as the optimal tissue for isolating protoplasts from soybean.

### 3.2 Effect of parameters on protoplast isolation efficiency from hypocotyls of soybean

To directly assess the effect of three parameters on soybean protoplast isolation efficiency, we performed shaking condition at 25 °C during enzymatic digestion, which is known to be optimal condition in previous studies (Wang et al., 2022; Han et al., 2023). First, the effect of enzyme digestion duration was evaluated based on the degree of hypocotyl decomposition (**Supplementary Figure S3**). The degree of hypocotyl decomposition increased depending on the enzyme digestion duration. However, the enzyme solution turned yellow after 18 h. This suggests that a prolonged enzyme digestion duration resulted in dissolution of impurities, which may negatively affect the viable protoplast yield. Therefore, the optimal enzyme digestion duration was determined to be 8–10 h.

**Figure 2:**
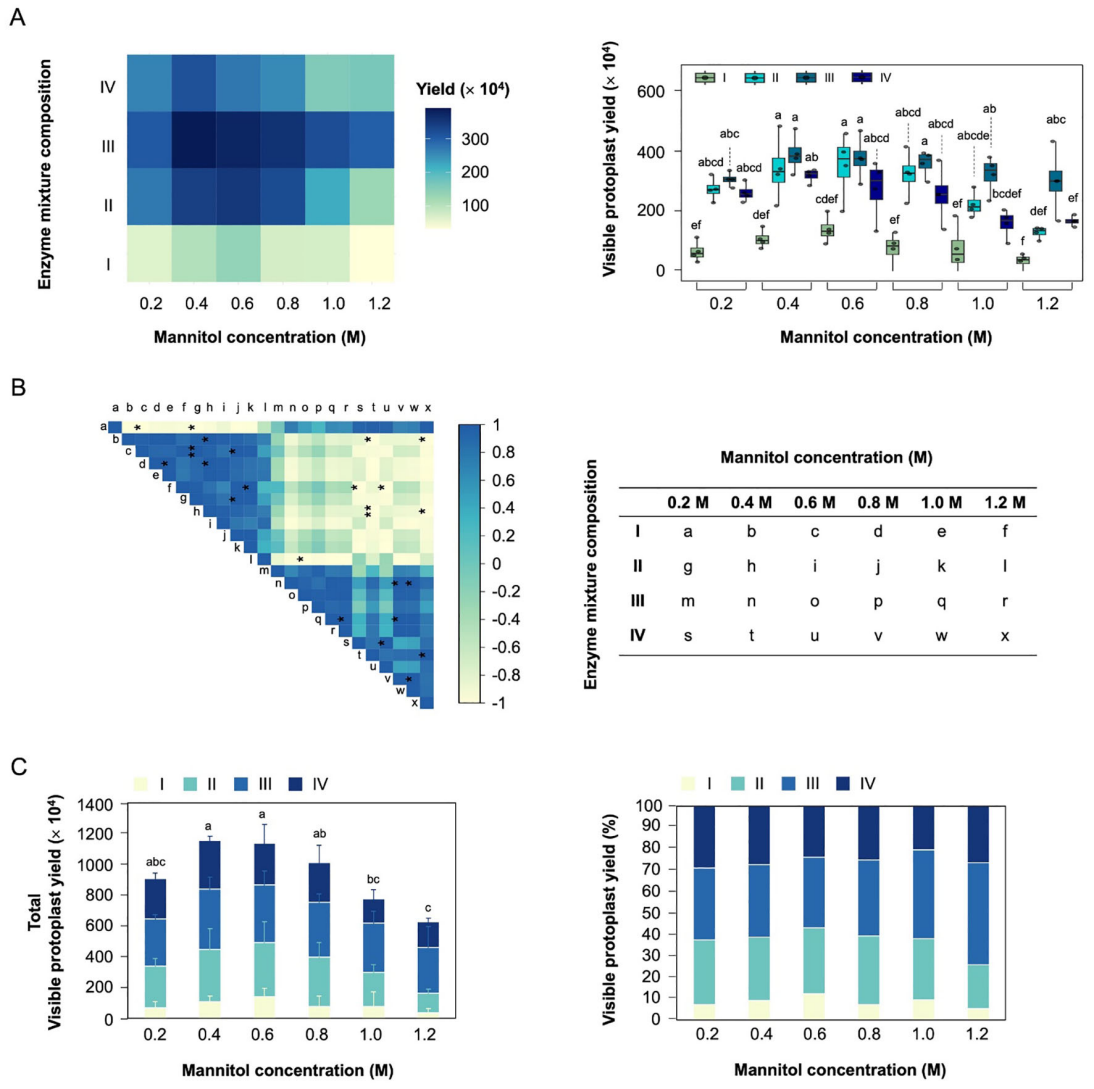
Optimization of visible protoplast yield for hypocotyl-derived protoplast isolation. **(A)** Effect of mannitol concentration and different enzyme mixture composition on visible protoplast yield. **(B)** Correlation plot showing the relationship between mannitol concentration and enzyme mixture composition. Colors correspond to the magnitude of Person correlation coefficients, as indicated by the color bar on the right, while asterisks denote the statistical significance of the correlations (*p < 0.05). **(C)** Stacked bar charts showing the total viable protoplast yield and the relative proportion of enzyme mixture composition at each mannitol concentration. The lowercase show the significant differences.

The effect of different mannitol concentration (0.2-1.2 M), enzyme mixture composition (**Supplementary Table S3**) on the viable protoplast yield were investigated with the hypocotyls of soybean (**Figure 2**). The viable protoplast yield remained consistently high in enzyme mixture composition III, regardless of mannitol concentration (**Figure 2A**). The enzyme mixture composition III and IV exhibited a stronger negative correlation with viable protoplast yield compared to enzyme mixture composition I and II (**Figure 2B**). However, the viable protoplast yield exhibited a decreasing trend with increasing mannitol concentration in enzyme mixture composition II and IV. These results suggests that enough cellulase, macerozyme, and viscozyme (enzyme mixture composition III) are essential to maximize viable protoplast yields. In addition, despite high concentration of cellulase, macerozyme, and viscozyme (enzyme mixture composition IV), high mannitol concentration (1.0-1.2 M) negatively affects the viable protoplast yield due to osmotic stress.

To determine the optimal parameters, we analyzed total viable protoplast yield in different enzyme mixture compositions and mannitol concentration (**Figure 2C**). The highest total viable protoplast yield was observed at 0.4-0.6 M mannitol concentration, with enzyme mixture solution III accounting for the largest proportion (33-34%). Especially, mannitol with enzyme mixture solution III achieved the highest viable protoplast yield (3.9 × 10^6^/g FW). These finding underscore the importance of maintain appropriate osmotic pressure and enzyme mixture composition to ensure the reliability of subsequent experiments.

### 3.3 Effect of parameters on PEG-Ca^2+^ mediated transfection efficiency in hypocotyl-derived protoplasts

**Figure 3:**
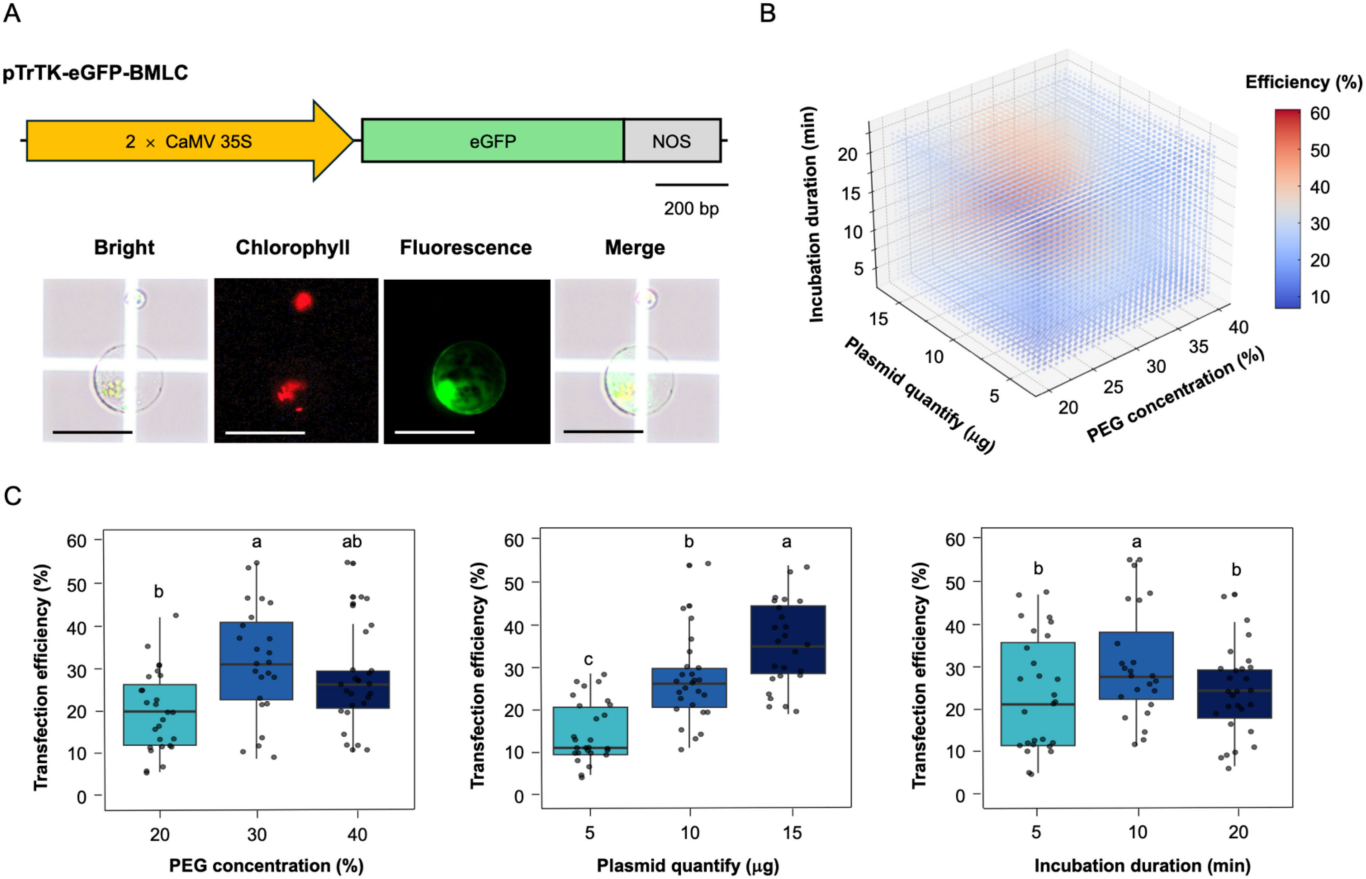
Optimization of parameters for protoplast transfection efficiency. **(A)** Assessment of transfection efficiency in hypocotyl-derived protoplasts. Top: the schematic diagram of the eGFP construction for transient expression. Bottom: eGFP microscopic images of transfected protoplasts. Scale bars = 50 μm. **(B)** Three-dimensional scatter plot showing the average transfection efficiency with variation in PEG concentration, plasmid quantify, and incubation duration. Colors correspond to the values of transfection efficiency, as indicated by the color bar on the right. **(C)** Effects of parameters on transfection efficiency. Left: effect of PEG concentration on the transfection efficiency. Middle: effect of plasmid quantity on the transfection efficiency. Right: effect of incubation duration on the transfection efficiency. The lowercase show the significant differences.

We also investigated the effect of different PEG concentration, plasmid DNA quantity, and PEG-Ca^2+^ incubation duration on transfection efficiency in hypocotyl-derived protoplasts (**Figure 3**). Determining appropriate parameters is crucial for maximizing transfection efficiency. To directly assess the effect of three parameters on soybean protoplasts transfection efficiency, we performed the PEG-Ca^2+^ mediated transfection with 2.5 × 10^4^ protoplasts based on the optimal condition (Yoo et al., 2007; Kang et al., 2023) and to mitigate the variable of plasmid volume, different concentration of plasmid DNA were employed, and different quantities of plasmid DNA were utilized within the same volume. This approach ensures consistency in the experimental condition and allows accurate assessment of the impact plasmid dosage on the experimental outcome. The success of the transfection was validated by introducing the pTrTK-eGFP-BMLC plasmid as a reporter into the hypocotyl-derived protoplasts and observing the fluorescence signal using Olympus BX53 fluorescence microscopy (**Figure 3A**). This method allows visualization and assessment of the transfection efficiency of gene delivery into the soybean protoplasts.

The 3-dimensional distribution visualizes the relationship between plasmid quantify, PEG concentration, and incubation duration in determining transfection efficiency (**Figure 3B**). The color gradient represents transfection efficiency, with blue indicating lower efficiency and red indicating higher efficiency. The optimal transfection condition appears to be at moderate PEG concentration, plasmid quantify, with an incubation duration of around 10 min. The transfection efficiency exhibited a significant positive correlation with increasing plasmid quantify. However, excessively PEG concentration and incubation duration negatively affect the transfection efficiency (**Figure 3C**). The highest transfection efficiency (61%) was reached at 30% (w/v) PEG concentration, 15 μg plasmid DNA quantities, and 10 min incubation duration. Based on the minimum requirement of 50% protoplast transfection efficiency necessary for obtaining reliable and reproducible experimental data in transient gene expression system (Yoo et al., 2007), these optimal parameters for PEG-Ca^2+^ transfection efficiency play a significant role in ensuring successful experimental outcomes and reliable data interpretation.

### 3.4 Validation of subcellular localization of target protein

**Figure 4:**
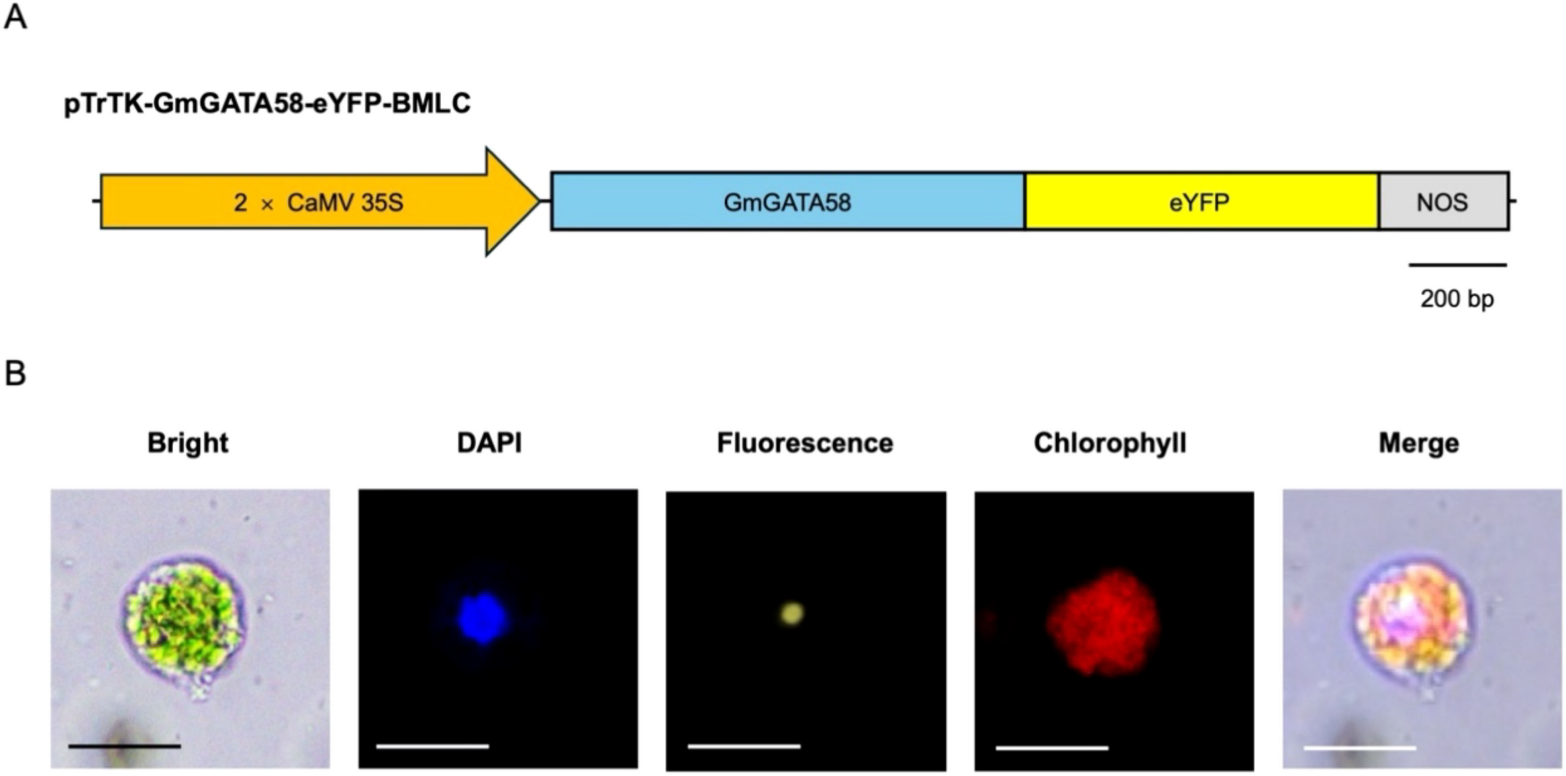
Subcellular localization of GmGATA58 in hypocotyl-derived protoplasts. **(A)** The schematic diagram of GmGATA58-eYFP construction for transient expression. **(B)** Microscopic images of subcellular localization of GmGATA58-eYFP in protoplast. DAPI is a nucleus location marker with blue signal. Chlorophyll is a chlorophyll autofluorescence in protoplast. DAPI, eYFP, and Chlorophyll fluorescence detection in the same protoplast. Scale bars = 50 μm.

To validate the protoplast transient gene expression system, we investigated whether the exogenously introduced gene can be targeted to the corresponding subcellular sites in hypocotyl-derived protoplasts (**Figure 4**). Hypocotyl-derived protoplasts were transfected with pTrTK-GmGATA58-eYFP-BMLC plasmid, linking the full-length CDS of GmGATA58 transcription factor in pTrTK-eYFP-BMLC plasmid (**Figure 4A**). PEG-Ca^2+^ mediated transfection was performed using the optimal transfection efficiency parameters, which included a 30% (w/v) PEG concentration, 15 μg plasmid quantify, and 10 min incubation duration. The 2 × CaMV 35S constitutive promoter-driven GmGATA58-eYFP induce gene expression in whole tissues of soybean. After incubation for 1 day, the results were observed with Olympus BX53 fluorescence microscopy. The fluorescence signal of transfected protoplasts with pTrTK-GmGATA58-eYFP-BMLC, were localized in the nucleus along with the DAPI signal (**Figure 4B**). These findings underscore the utility of the transient gene expression system in hypocotyl-derived protoplast for subcellular localization analysis.

### 3.5 Validation of recombinant protein transfection system using GUS/NAN protein activity

**Figure 5:**
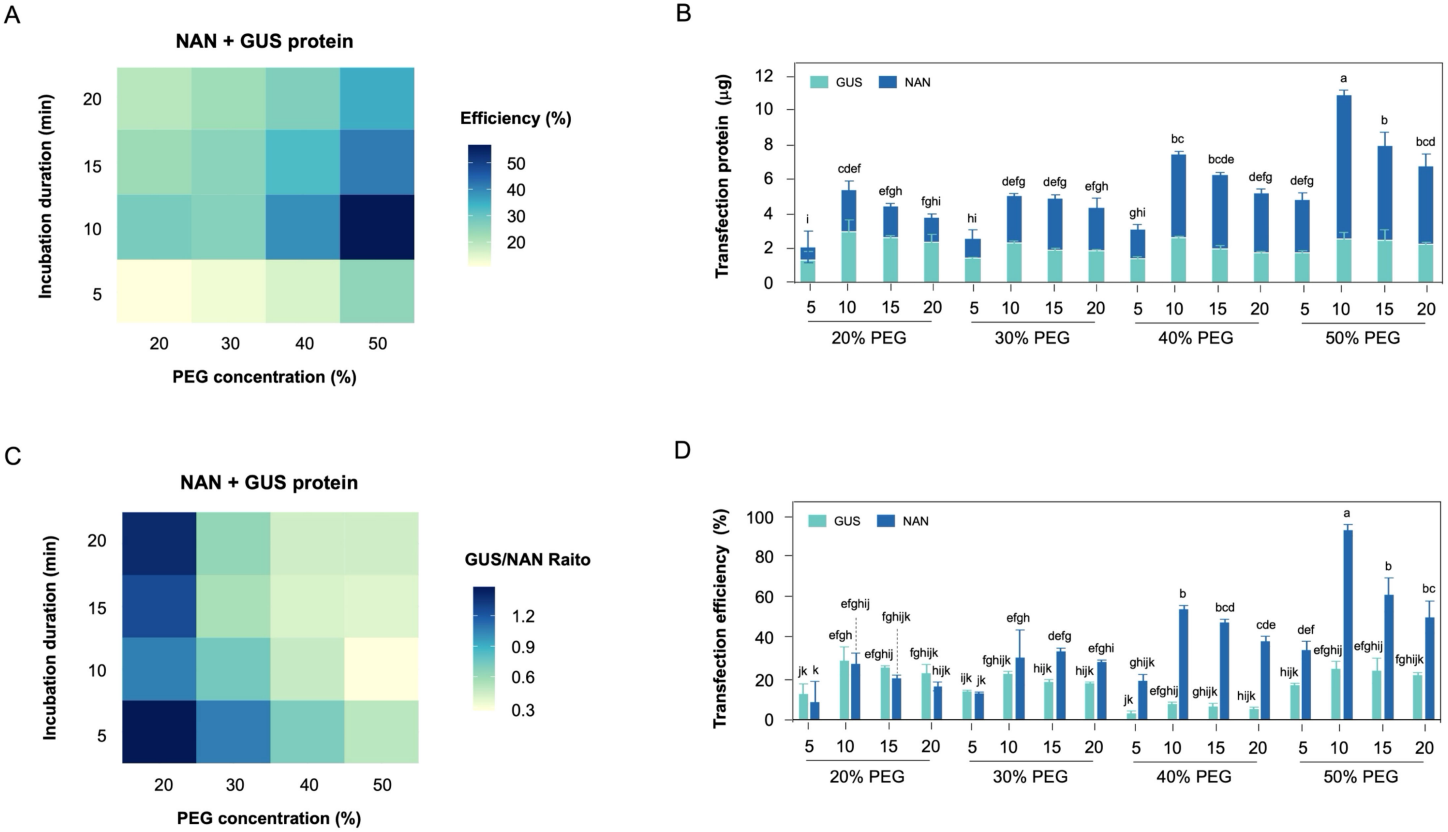
Establishment of recombinant protein co-transfection in hypocotyl-derived protoplasts. **(A)** The heatmap of protein co-transfection efficiency under PEG concentration and incubation duration. **(B)** Stacked bar charts showing the total transfection recombinant protein in protoplast. **(C)** The heatmap of each recombinant protein transfection efficiency ratio under PEG concentration and incubation duration. **(D)** Effect of PEG concentration and incubation duration on the transfection efficiency ratio of each recombinant protein.

We also investigated the probability of suitable recombinant protein transfection system in hypocotyl-derived protoplasts (**Figure 5**). GUS and NAN protein are widely utilized due to their high sensitivity and ease of quantification in cell-free extracts (Jefferson, 1987; Kirby and Kavanagh, 2002). Although both enzymes share the common characteristic of utilizing methylumbelliferone-based substrates, NAN protein specifically cleaves *α*-glycosidic bond of 4-MU-NANANa, whereas GUS protein cleaves *β*-glycosidic bond of 4-MUG. Theses substrate specificity make GUS protein and NAN protein ideal partners for simultaneous applications.

The co-transfection efficiency of each recombinant protein tended to increase with higher PEG concentration, and higher co-transfection efficiency observed at 10 min incubation duration (**Figure 5A**). The co-transfection amount of each recombinant protein revealed that recombinant His_6_-NAN protein exhibited a higher transfection amount compared to recombinant His_6_-GUS protein (**Figure 5B**). In particular, a significant difference in incubation duration was observed at 40-50% (w/v) PEG concentration compared to other PEG concentration. Interestingly, the transfection ratio of recombinant GUS/NAN was confirmed that each recombinant protein was transfected at the same rate at lower PEG concentration (**Figure 5C**). Theses result suggests that the transfection efficiency of recombinant His_6_-NAN protein, which has a smaller protein size, tended to decrease with lower PEG concentration (**Figure 5D**), indicating that the transfection efficiency of smaller protein is more sensitive to PEG concentration. In addition, the incubation duration was consistently observed the optimal transfection efficiency at 10 min and tended to decrease over time. Theses finding suggests that the prolonged exposure to PEG negatively affects protein stability.

## 4 Discussion

Much research efforts are underway to augment soybean productivity due to the agricultural and economic significance of soybean. Also, Plant protoplast-based systems encompass high-throughput analysis of gene function through transient gene expression (Xiong et al., 2019; Kim et al., 2022), genetic manipulation facilitated by CRISPR/Cas9 RNP-mediated genome editing (Svitashev et al., 2016; Liang et al., 2018; Subburaj et al., 2022), and the investigation of cell-to-cell interaction by single-cell transcriptomics (Giacomello, 2021; Ryu et al., 2021). These systems are emerging as alternative to circumvent the constrains associated with traditional plant transformation. Currently, successful protoplast isolation has been achieved in various plant models and non-model species (Reed and Bargmann, 2021). However, much research is still aimed to address prevalent challenges, including low protoplast yield and viability attributable to the distinct physiological traits of individual plant species. In particular, investigations into enhancing protoplast yield and transfection efficiency in soybean remain still limit. Consequently, the establishment of efficient and universally applicable protoplast isolation and transfection in soybean is essential experimental prerequisites for the study of regulatory mechanisms through genetic functional analysis of soybean.

In this study, we validated the viable protoplast yield of different tissues in soybean seedlings [*Glycine max* (L.) Merr.] cultivar (Williams 82) through FDA staining (**Figure 1**, **Supplementary Figure S1**). The epicuticular wax of soybean leaves plays a crucial role in preventing pathogen infection and reducing water loss. Interestingly, the epicuticular wax of leaves is highly hydrophobic to water (Damato et al., 2017), which supports the observation that enzymatic digestion is minimal in unifoliate leaves.

Optimal conditions for protoplast isolation from hypocotyls of 2-week-old Williams 82 were determined by assessing key parameters, including mannitol concentration, enzyme mixture composition, and enzymatic digestion duration (**Figure 2**, **Supplementary Figure S2**). Mannitol serves to regulate osmotic pressure inside and outside the plant protoplasts whose cell walls have been removed, thereby maintain protoplast stability and enhancing the efficiency of enzyme treatment. Moreover, enzyme mixture composition includes cellulase and macerozyme, which are responsible for breaking down cellulose, pectin, and other cell wall components in plant cell walls to release protoplasts. The optimal condition for viable protoplast yield were achieved with 0.4 M mannitol concentration, enzyme mixture containing the combination of 1.5% (w/v) Cellulase R-10 and 0.4% (w/v) Macerozyme R-10 for 8 h enzymatic digestion duration. The suggested optimal condition ensured high viable protoplast yields (>3.0 × 10^6^/g FW). Compared to previously reported protocols utilizing leaf tissues for soybean protoplast isolation (Wu and Hanzawa, 2018), which employed young unifoliate leaves for protoplast preparation and transient gene expression analyses, our hypocotyl-based approach offers several advantages. Hypocotyl tissues provide developmental uniformity and reduced heterogeneity in cell wall composition, leading to improved reproducibility and protoplast viability. Additionally, our method employs a simplified and cost-effective enzyme composition without compromising yield or viability. These improvements address the limitations observed in earlier protocols and enhance the efficiency of protoplast isolation in soybean.

Optimal transfection of hypocotyl-derived protoplasts was determined by assessing key parameters, including PEG concentration, plasmid DNA quantity, and PEG-Ca^2+^ incubation duration using transient gene expression system (**Figure 3**). PEG 4000 (PEG with 4000 molecular weight) can destabilize protoplast by disrupting the phospholipid bilayer of the junctional membrane (Kao and Michayluk, 1974). Moreover, plasmid DNA quantity can impact protoplast stability as traverses the protoplast’s phospholipid bilayer membrane (Armstrong et al., 1990; Kang et al., 2023). The transfection efficiency was evaluated using a transient gene expression system targeting expression of eGFP fluorescent protein (**Figure 3**). The optimal PEG-Ca^2+^ mediated transfection efficiency was achieved with 15 μg of plasmid in 30% (w/v) PEG-Ca^2+^ concentration for 10 min incubation duration.

To evaluate the applicability of the transient gene expression system, subcellular localization was performed to confirm whether the target protein was in the corresponding subcellular site of hypocotyl-derived protoplasts (**Figure 4**). It was demonstrated that the DAPI, which penetrates the cell membrane and binds DNA within the cell nucleus to specify the location the cell nucleus, overlapped with the fluorescent signal of the GmGATA58 transcription factor, which is known to localize in the cell nucleus (Zhang et al., 2015, 2020). Moreover, we validated the recombinant GUS/NAN protein co-transfection efficiency, which is essential for CRISPR/Cas RNP-mediated gene editing in protoplast (**Figure 5**). Purified recombinant protein tend to exhibit higher transfection efficiency at higher PEG concentration as protein size decreases. Additionally, a 10 min incubation duration results in high transfection efficiency regardless of protein size. Given the molecular weight of the recombinant Cas9 protein for CRISPR/Cas9 RNP-mediated gene editing is 166.8 kDa, a 20% (w/v) PEG concentration is expected to achieve the highest transfection efficiency during a 10 min incubation duration. Therefore, the protoplast yield and viability under the optimal conditions outlined in this study are sufficient to fulfill the requirements for protoplast-based transformation and various associated experiments.

## 5 Conclusion

The establishment of protoplast isolation and PEG-Ca^2+^ mediated transfection in soybean offers avenues for comprehensively elucidating the genetic functions and regulatory mechanisms, facilitating targeted improvements in plant traits for agricultural benefit. In this study, we addressed the limitations of dependence on viable protoplast and transfection efficiency for transient expression by establishing conditions for high-yield protoplast isolation and PEG-Ca^2+^ mediated transfection. Our research can be successfully applied to investigate into gene function and soybean physiological molecular mechanisms and can also be extended to CRISPR/Cas RNP-mediated genome editing and single-cell transcriptomics.

## Data Availability

The datasets presented in this study can be found in online repositories. The names of the repository/repositories and accession number(s) can be found in the article/**Supplementary Material**.

## Supplementary material

Supplementary Figure 1 Viability assessment of soybean protoplasts and its correlation with absorbance (OD _680 nm_). **(A)** Microscopic images of soybean protoplasts stained with fluorescein diacetate (FDA). Left: Bright-field image showing protoplasts. Middle: Fluorescence image indicating viable protoplasts stained with FDA (green fluorescence). Right: Merged image of bright-field and fluorescence. Scale bars = 100 μm. **(B)** Correlation between absorbance (OD_680 nm_) and visible protoplast yield. A strong linear correlation was observed (R2 = 0.9887), indicating that absorbance can be used to estimate visible protoplasts yield.

Supplementary Figure 2 The SDS-PAGE of the purified recombinant His6-NAN and His6-GUS protein.

Supplementary Figure 3 Time-course enzymatic digestion efficiency of hypocotyl from soybean seedlings. Soybean hypocotyl tissues (1 g fresh weight) were subjected to enzymatic digestion using Enzyme solution 3 (0.4 M mannitol, 1.5% (w/v) cellulase, 0.4% (w/v) macerozyme, and 1% (w/v) viscozyme).

Supplementary Table 1 The composition of protoplast isolation solution used in this study.

Supplementary Table 2 List of primers used in this study.

Supplementary Table 3 Different enzyme mixture composition used on protoplast isolation.
