# Supplementary material for: Establishment of efficient hypocotyl-derived protoplast isolation and its application in soybean (Glycine max [L.] Merr.)
Source: Front Plant Sci. 2025 May 20;16:1587927. doi: 10.3389/fpls.2025.1587927 (PMC12130014; doi:10.3389/fpls.2025.1587927)
Supplement: Supplementary Figure 1 — Viability assessment of soybean protoplasts and its correlation with absorbance (OD 680 nm). (A) Microscopic images of soybean protoplasts stained with fluorescein diacetate (FDA). Left: Bright-field image showing protoplasts. Middle: Fluorescence image indicating viable protoplasts stained with FDA (green fluorescence). Right: Merged image of bright-field and fluorescence. Scale bars = 100 μm. (B) Correlation between absorbance (OD680 nm) and visible protoplast yield. A strong linear correlation was observed (R2 = 0.9887), indicating that absorbance can be used to estimate visible protoplasts yield. [file DataSheet1.docx]

**Supplementary data**

**Supplementary Figure S1. Viability assessment of soybean protoplasts and its correlation with absorbance (OD _680 nm_). (A)** Microscopic images of soybean protoplasts stained with fluorescein diacetate (FDA). Left: Bright-field image showing protoplasts. Middle: Fluorescence image indicating viable protoplasts stained with FDA (green fluorescence). Right: Merged image of bright-field and fluorescence. Scale bars = 100 μm. (**B**) Correlation between absorbance (OD_680 nm_) and visible protoplast yield. A strong linear correlation was observed (R2 = 0.9887), indicating that absorbance can be used to estimate visible protoplasts yield.

**Supplementary Figure S2. The SDS-PAGE of the purified recombinant His_6_-NAN and His_6_-GUS protein.**

**Supplementary Figure S3. Time-course enzymatic digestion efficiency of hypocotyl from soybean seedlings.** Soybean hypocotyl tissues (1 g fresh weight) were subjected to enzymatic digestion using Enzyme solution 3 (0.4 M mannitol, 1.5% (w/v) cellulase, 0.4% (w/v) macerozyme, and 1% (w/v) viscozyme).

**Supplementary Figure S1. Viability assessment of soybean protoplasts and its correlation with absorbance (OD _680 nm_). (A)** Microscopic images of soybean protoplasts stained with fluorescein diacetate (FDA). Left: Bright-field image showing protoplasts. Middle: Fluorescence image indicating viable protoplasts stained with FDA (green fluorescence). Right: Merged image of bright-field and fluorescence. Scale bars = 100 μm. (**B**) Correlation between absorbance (OD_680 nm_) and visible protoplast yield. A strong linear correlation was observed (R2 = 0.9887), indicating that absorbance can be used to estimate visible protoplasts yield.

**Supplementary Figure S2. The SDS-PAGE of the purified recombinant His_6_-NAN and His_6_-GUS protein.**


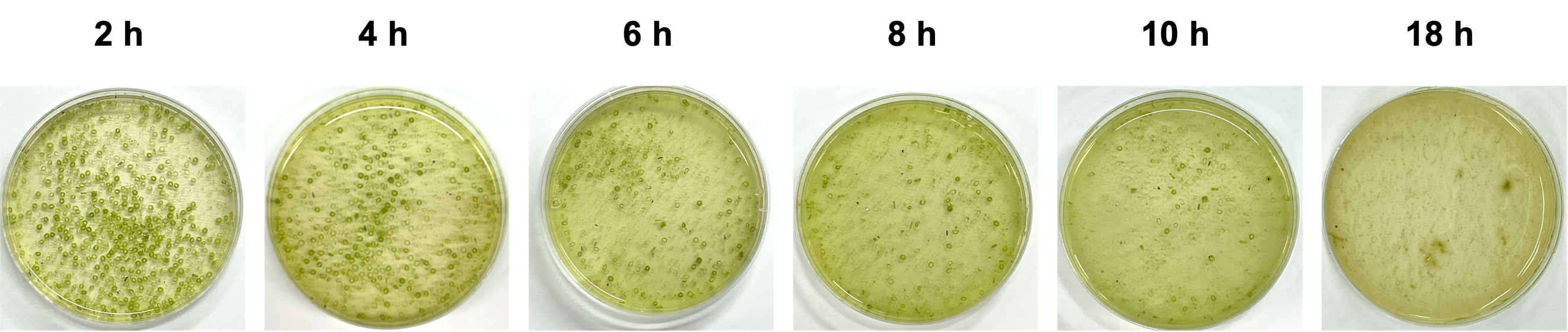


**Supplementary Figure S3. Time-course enzymatic digestion efficiency of hypocotyl from soybean seedlings.** Soybean hypocotyl tissues (1 g fresh weight) were subjected to enzymatic digestion using Enzyme solution 3 (0.4 M mannitol, 1.5% (w/v) cellulase, 0.4% (w/v) macerozyme, and 1% (w/v) viscozyme).
