# Supplementary material for: Establishment of efficient hypocotyl-derived protoplast isolation and its application in soybean (Glycine max [L.] Merr.)
Source: Front Plant Sci. 2025 May 20;16:1587927. doi: 10.3389/fpls.2025.1587927 (PMC12130014; doi:10.3389/fpls.2025.1587927)

**Supplementary data**

**Supplementary Table S1.** The composition of protoplast isolation solution used in this study

**Supplementary Table S2**. List of primers used in this study

**Supplementary Table S3**. Different enzyme mixture composition used on protoplast isolation

**Supplementary Table S1.** The composition of protoplast isolation solution used in this study


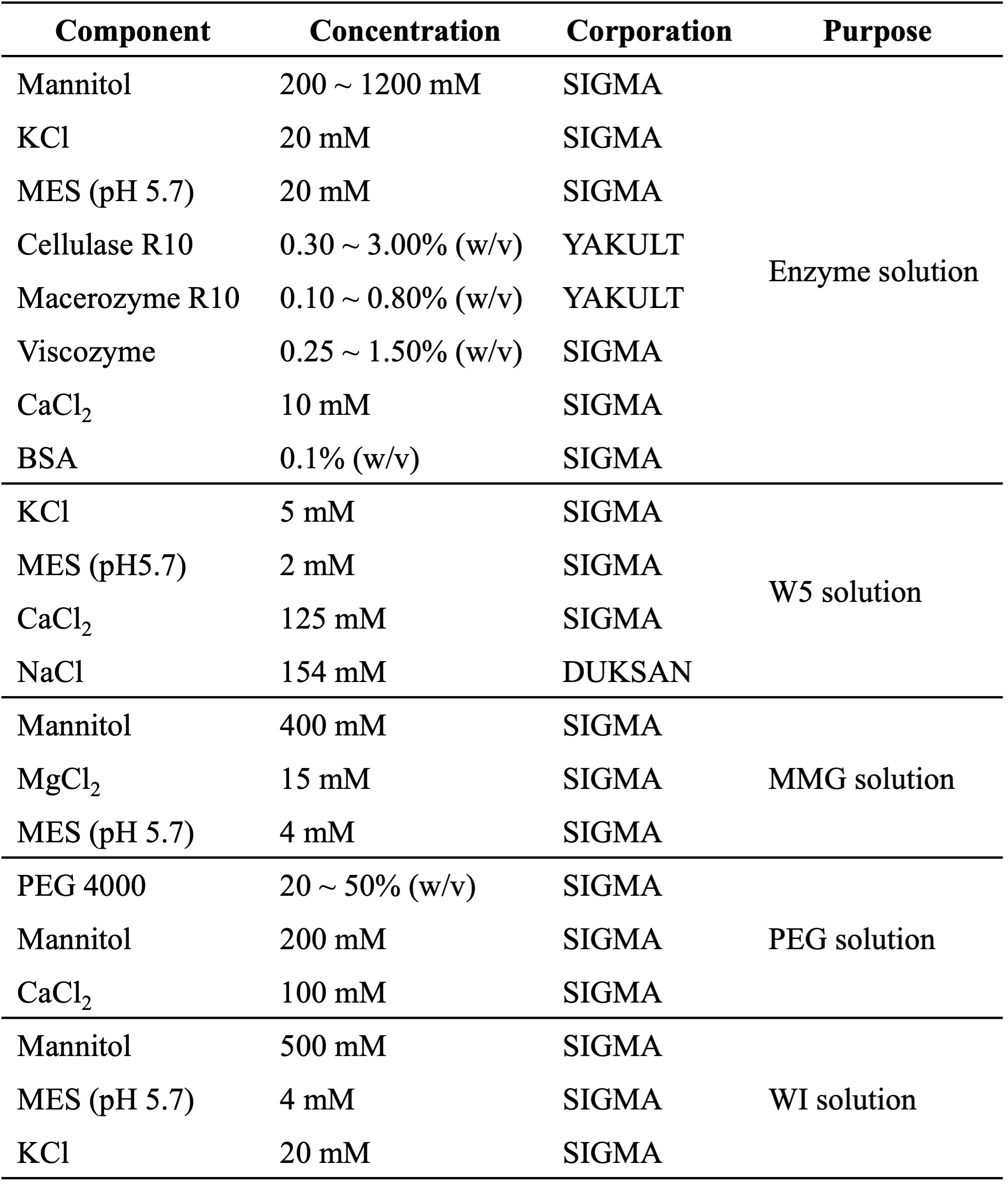


**Supplementary Table S2**. List of primers used in this study

**Supplementary Table S3**. Different enzyme mixture composition used on protoplast isolation


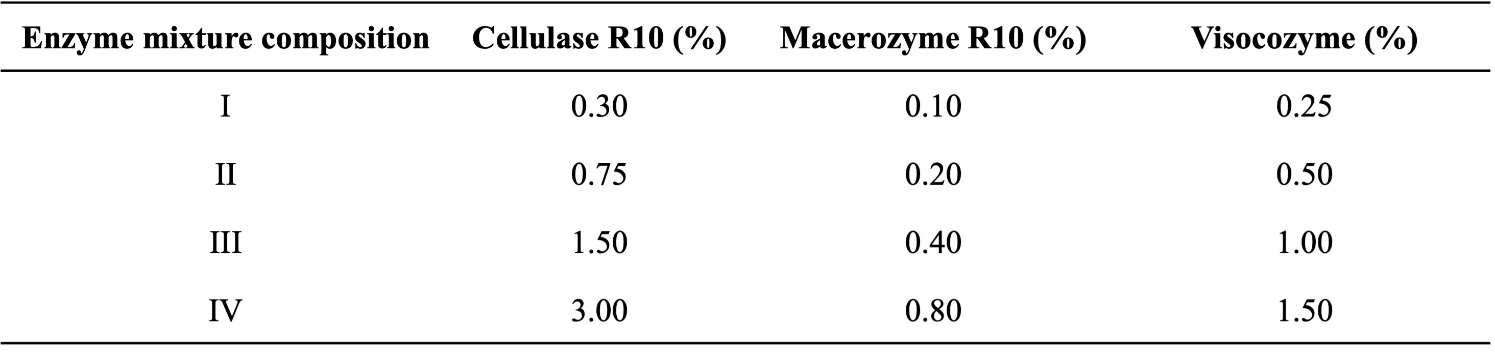

Supplement: Supplementary Table 1 — The composition of protoplast isolation solution used in this study. [file Table1.docx]
